# Supplementary material for: Subpopulation-specific machine learning prognosis for underrepresented patients with double prioritized bias correction
Source: Commun Med (Lond). 2022 Sep 1;2:111. doi: 10.1038/s43856-022-00165-w (PMC9436942; doi:10.1038/s43856-022-00165-w)
Supplement: Supplementary file 1 — Supplementary Information [file 43856_2022_165_MOESM1_ESM.pdf]

Supplementary Information

Subpopulation-specific Machine Learning Prognosis for Underrepresented Patients with Double Prioritized Bias Correction

Sharmin Afrose[1*], Wenjia Song[1*], Charles B. Nemeroff[2], Chang Lu[3], Danfeng (Daphne) Yao[1#]

[1]Department of Computer Science, Virginia Tech, Blacksburg, VA, USA. [2]Department of Psychiatry and Behavioral Sciences, the University of Texas at Austin Dell Medical School, Austin, TX, USA. [3]Department of Chemical Engineering, Virginia Tech, Blacksburg, VA, USA.

[*]Contributed equally

[#]Corresponding Author: Danfeng (Daphne) Yao, Department of Computer Science, Virginia Tech, Blacksburg, VA 24060, USA. danfeng@vt.edu; +1 (540) 231-7787

Supplementary Note

BCS Class 1: Patient does not survive more than 5 years after breast cancer diagnosis;
IHM Class 1: Based on the first 48 hours of ICU information, the patient dies in ICU
LCS Class 1: Patient survives more than 5 years after lung cancer diagnosis
Decomp Class 1: Patient's health deteriorates after 24 hours

$$\text{Recall C1 or Sensitivity} = \frac{\#\ Predicted\ True\ \ Class\ 1}{\#\ True\ Class\ 1} \tag{1}$$

$$\text{Recall C0 or Specificity} = \frac{\#\ Predicted\ True\ Class\ 0}{\#\ True\ Class\ 0} \tag{2}$$

$$\text{Precision C1 or Positive Predictive Value} = \frac{\#\ Predicted\ True\ Class\ 1}{\#\ Predicted\ Class\ 1} \tag{3}$$

$$\text{Precision C0 or Negative Predictive Value} = \frac{\#Predicted\ True\ Class\ 0}{\#\ Predicted\ Class\ 0} \tag{4}$$

$$\text{Accuracy} = \frac{\#\ Predicted\ True\ Class\ 1 + \#\ Predicted\ True\ Class\ 0}{\#True\ Class\ 1\ +\ \#True\ Class\ 0} \tag{5}$$

$$\text{Balanced Accuracy} = \frac{Recall\ C1 + Recall\ C0}{2} \tag{6}$$

$$\text{F1-Score C1} = 2 * \frac{Precision\ C1 * Recall\ C1}{Precision\ C1 + Recall\ C1} \tag{7}$$

$$\text{F1-Score C0} = 2 * \frac{Precision\ C0 * Recall\ C0}{Precision\ C0 + Recall\ C0} \tag{8}$$

$$MCC = \frac{\#Predicted\ True\ Class\ 1 \times \#Predicted\ True\ Class\ 0 - \#Predicted\ False\ Class\ 1 \times \#Predicted\ False\ Class\ 0}{\sqrt{\#Predicted\ Class\ 1 \times \#True\ Class\ 1 \times \#Predicted\ Class\ 0 \times \#True\ Class\ 0}} \tag{9}$$

Supplementary Figures and Tables

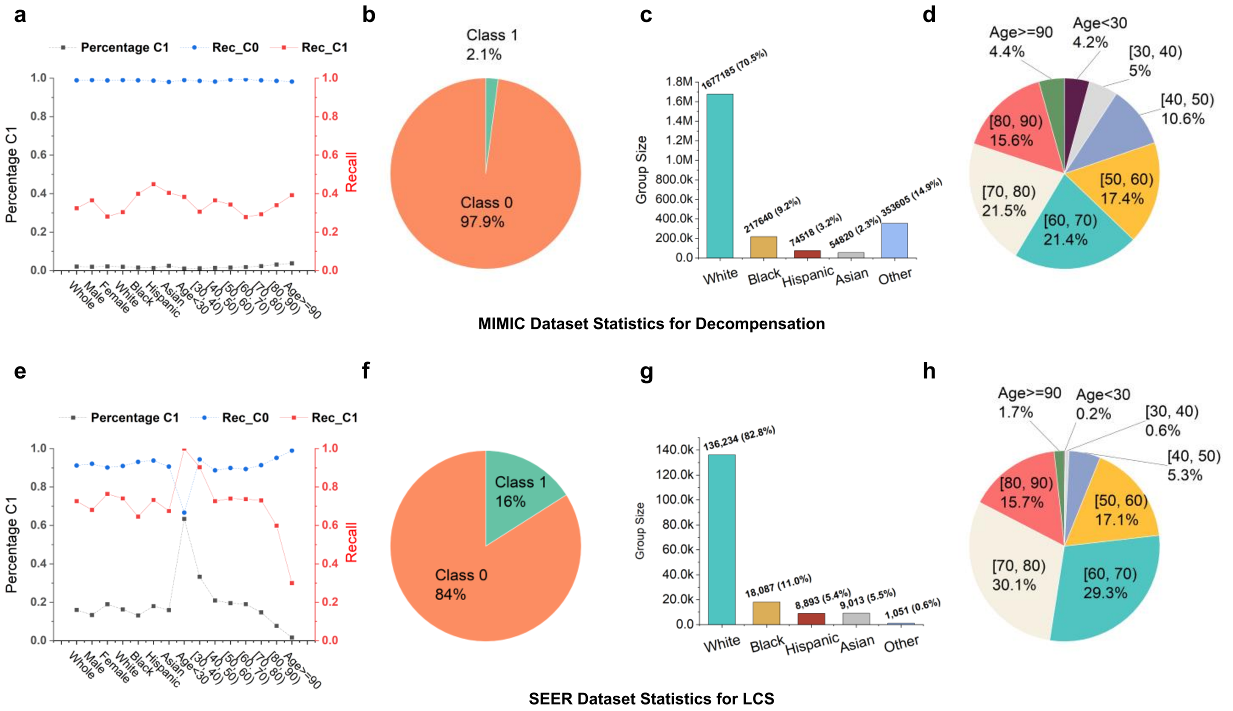

Supplementary Figure 1: Recall values for both classes C0 and C1 and training data statistics for the decompensation and the 5-year lung cancer survivability (LCS) tasks. (a) Percentage of the minority class C1, Recall C0, and Recall C1 of each subgroup of the MIMIC dataset for the Decomp task. Statistics of **(b)** prediction class distribution, **(c)** racial group distribution, and **(d)** age group distribution for the MIMIC Decomp dataset. The MIMIC Decomp training set consists of 44.3% female samples and 55.7% male samples. **(e)** Percentage of the minority class C1, Recall C0, and Recall C1 of each subgroup of the SEER dataset for the LCS task. Statistics of **(f)** prediction class distribution, **(g)** racial group distribution, and **(h)** age group distribution for the SEER LCS dataset. The SEER LCS training set consists of 47.0% female samples and 53.0% male samples.

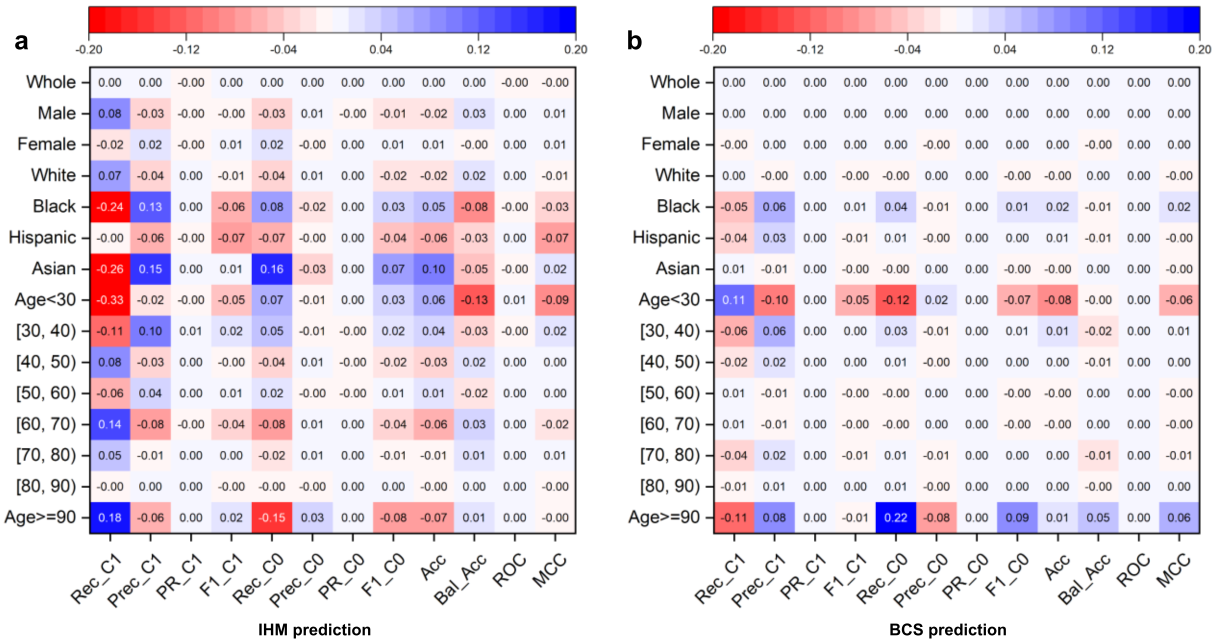

Supplementary Figure 2: Differences in performance of the original machine learning models (no bias correction) using subgroup thresholds (i.e., different optimized thresholds for different demographic groups) and using the whole group threshold. Positive values mean that using a subgroup optimized threshold improves the performance. Rec_C1, Prec_C1, PR_C1, F1_C1, Rec_C0, Prec_C0, PR_C0, F1_C0, Acc, Bal_Acc, ROC, MCC stand for Recall Class 1, Precision Class 1, Area Under the Precision-Recall Curve Class 1, F1 score Class 1, Recall Class 0, Precision Class 0, Area Under the Precision-Recall Curve Class 0, F1 score Class 0, Accuracy, Balanced Accuracy, Area under the ROC Curve, Matthews Correlation Coefficient, respectively. The performance differences between the two settings are shown for **(a)** the IHM prediction and **(b)** the BCS prediction.

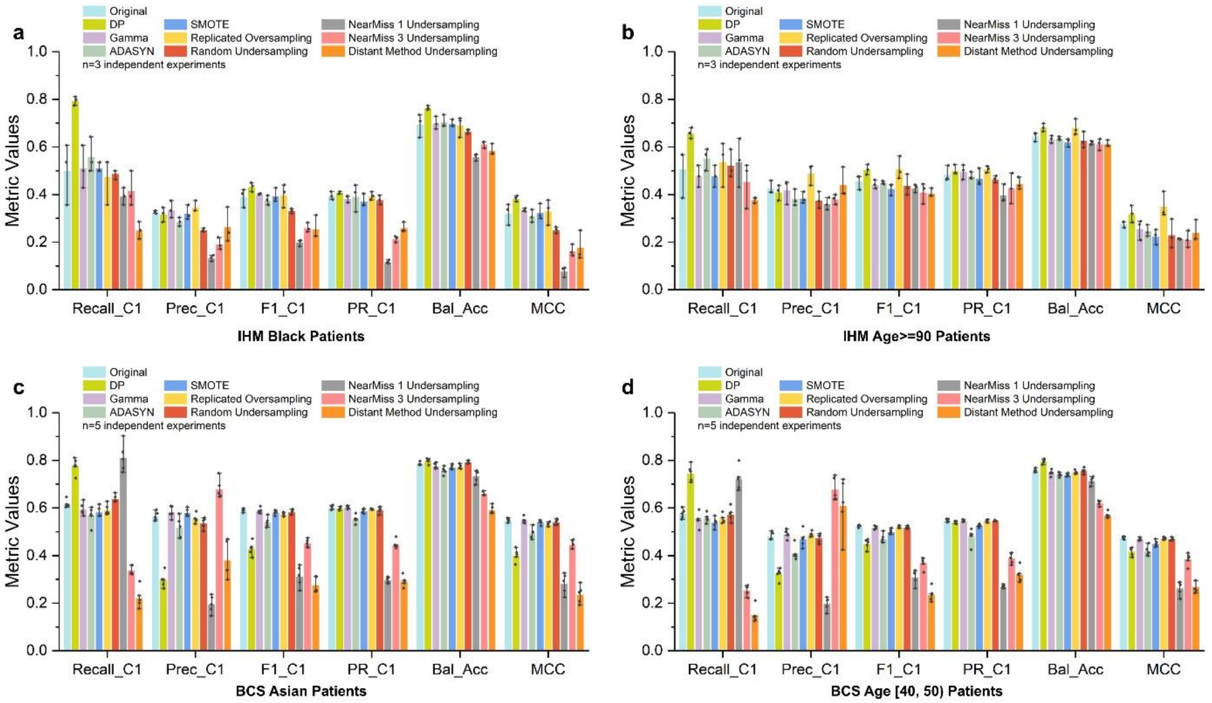

Supplementary Figure 3: In-hospital mortality (IHM) prediction and 5-year breast cancer survivability (BCS) prediction under various sampling conditions, including DP and the original machine learning model without any sampling, in terms of minority class recall, precision, F1 score, AUC-PR, balanced accuracy, and Matthews Correlation Coefficient (MCC). Prediction results from the original model and different sampling models for **(a)** Black patients and **(b)** age>=90 patients in the IHM prediction with the MIMIC III dataset. Prediction results from the original model and different sampling models for **(c)** Asian patients and **(d)** age [40, 50) patients in the BCS prediction with the SEER dataset.

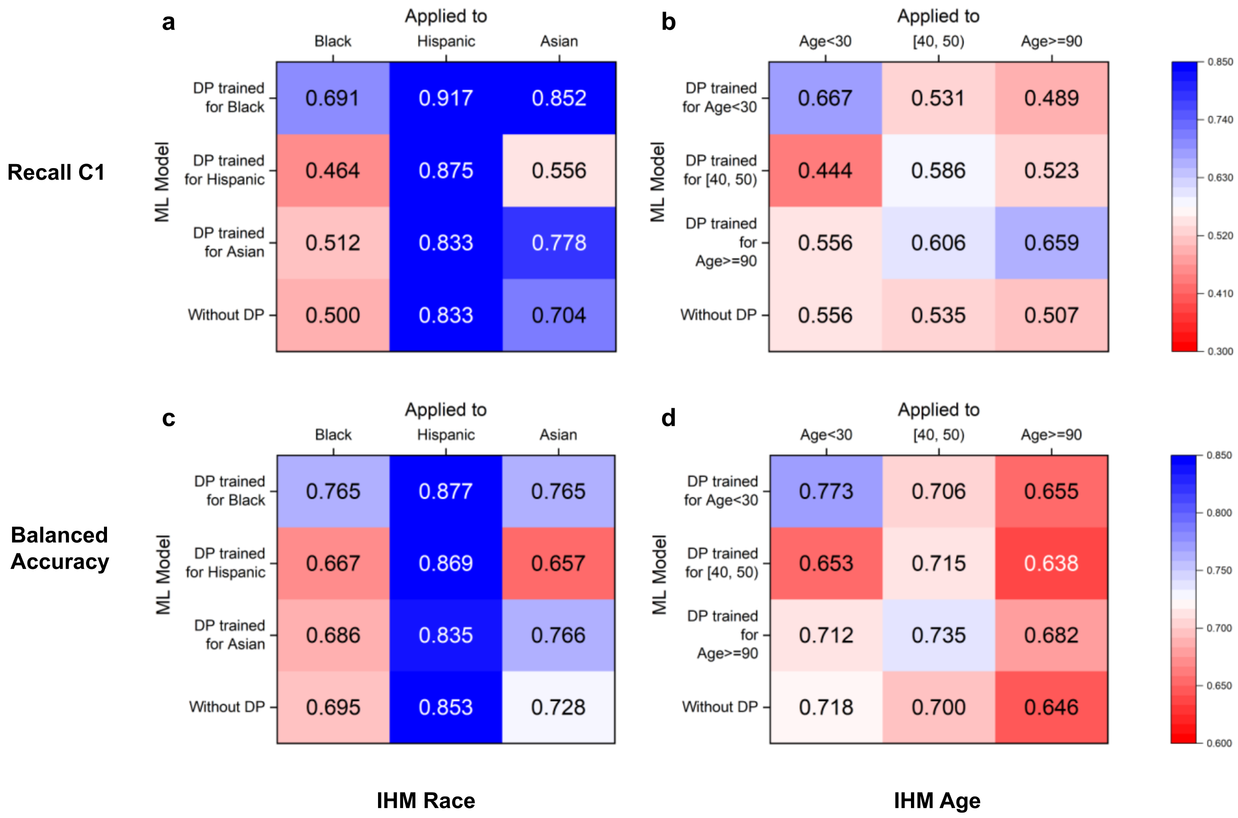

Supplementary Figure 4: DP's cross-group performance under various race and age settings for recall C1 and balanced accuracy for the IHM prediction. In subfigures, each row corresponds to a DP model trained for a specific subgroup. Each column represents a subgroup that a model is evaluated on. The values on the diagonal are the performance of a matching DP model, i.e., a DP model applied to the subgroup that it is designed for. The last rows show the group's performance in the original model. To prevent overfitting, our method chooses optimal thresholds based on whole group performance. DP cross-group performance in terms of recall C1 for **(a)** race subgroups and **(b)** age subgroups for the IHM prediction. DP cross-group performance in terms of balanced accuracy for **(c)** race subgroups and **(d)** age subgroups for the IHM prediction.

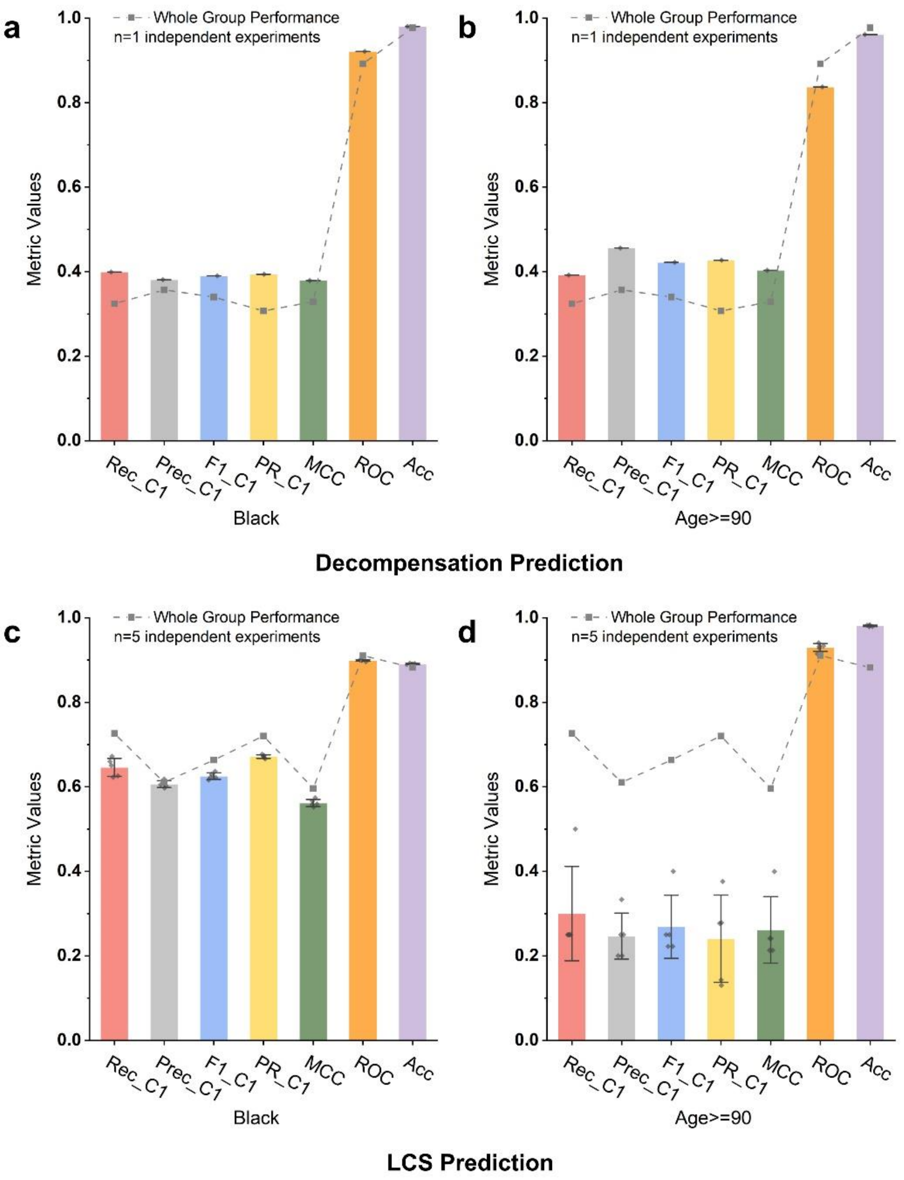

Supplementary Figure 5: Comparison of whole-population metrics with minority-class-specific metrics. Some whole-population metrics (e.g., AUC ROC and accuracy) are misleading for the minority class. These deceptive metrics show high values, whereas the prediction is weak for the minority class. **(a)** Black subgroup performance for decompensation prediction. **(b)** Age 90+ subgroup performance for decompensation prediction. **(c)** Black subgroup performance for LCS prediction. **(d)** Age 90+ subgroup performance for LCS prediction. Due to the slow decompensation computation, each decompensation prediction is executed only once.

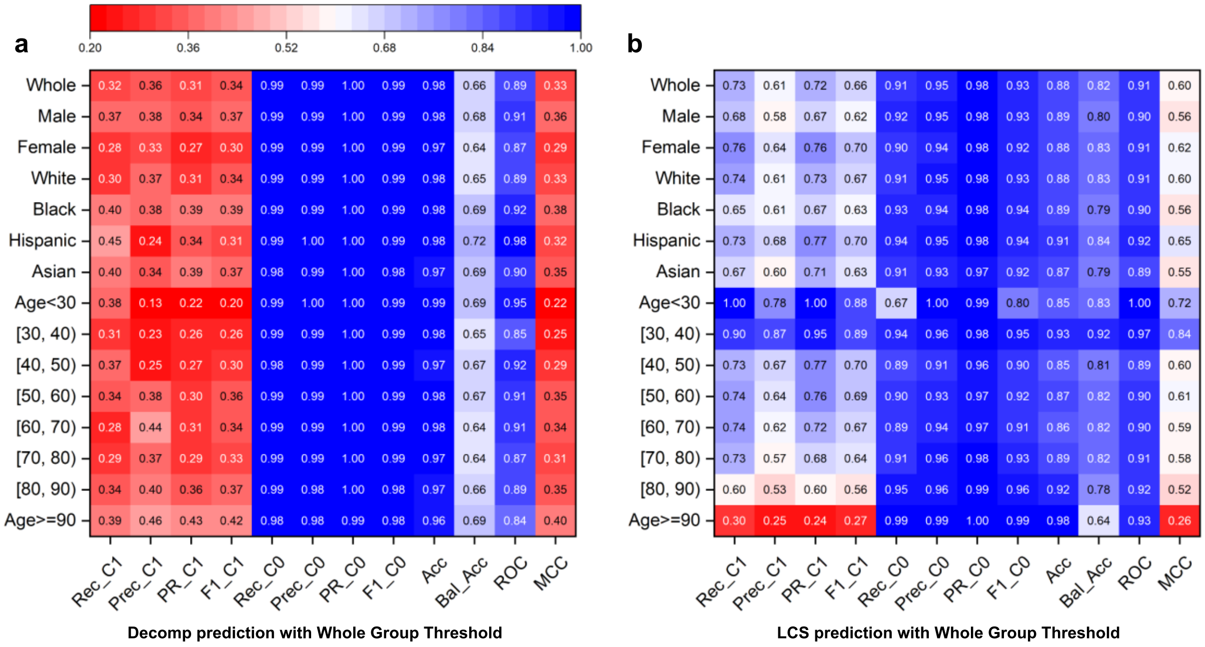

Supplementary Figure 6: Prediction results under the original machine learning models (no bias correction) using one optimized threshold for all demographic groups. Rec_C1, Prec_C1, PR_C1, F1_C1, Rec_C0, Prec_C0, PR_C0, F1_C0, Acc, Bal_Acc, ROC, MCC stand for Recall Class 1, Precision Class 1, Area Under the Precision-Recall Curve Class 1, F1 score Class 1, Recall Class 0, Precision Class 0, Area Under the Precision-Recall Curve Class 0, F1 score Class 0, Accuracy, Balanced Accuracy, Area under the ROC Curve, Matthews Correlation Coefficient (MCC), respectively. **(a)** Prediction results for the decompensation prediction. The minority Class 1 represents patients whose health deteriorates after 24 hours. **(b)** Prediction results for the Lung cancer survivability (LCS) prediction. The minority Class 1 represents patients who survive lung cancer for at least 5 years after the diagnosis.

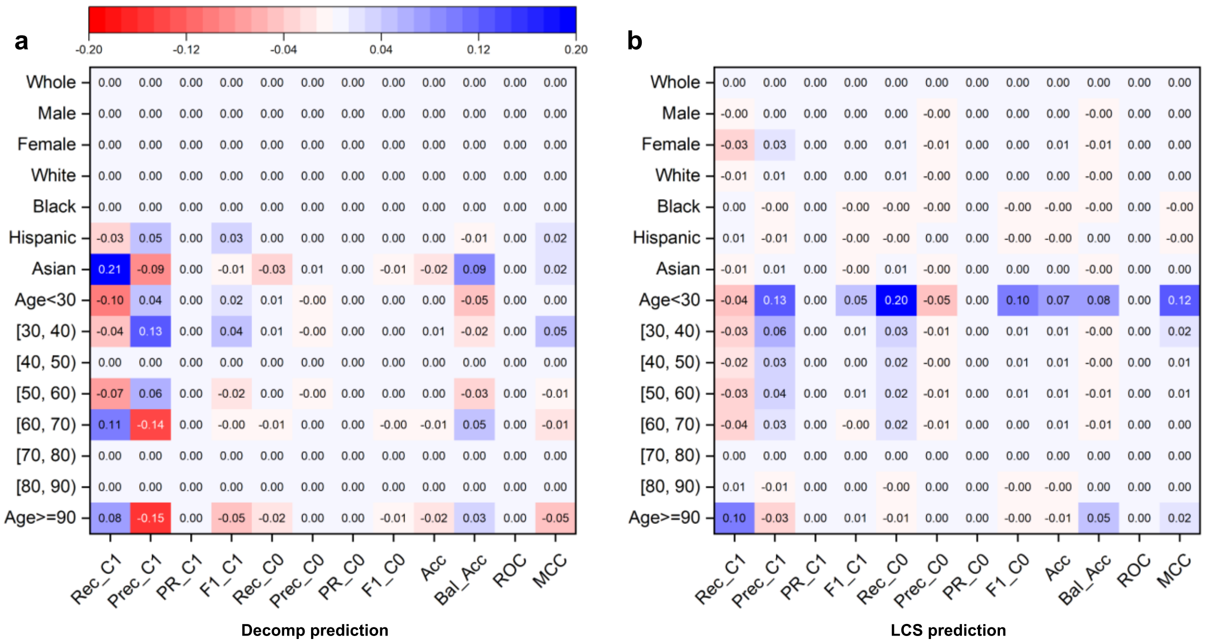

Supplementary Figure 7: Differences in performance of the original machine learning models (no bias correction) using subgroup thresholds (i.e., different optimized thresholds for different demographic groups) and using the whole group threshold. Positive values mean that using a subgroup optimized threshold improves the performance. Rec_C1, Prec_C1, PR_C1, F1_C1, Rec_C0, Prec_C0, PR_C0, F1_C0, Acc, Bal_Acc, ROC, MCC stand for Recall Class 1, Precision Class 1, Area Under the Precision-Recall Curve Class 1, F1 score Class 1, Recall Class 0, Precision Class 0, Area Under the Precision-Recall Curve Class 0, F1 score Class 0, Accuracy, Balanced Accuracy, Area under the ROC Curve, and Matthews Correlation Coefficient (MCC), respectively. The performance differences between the two settings for **(a)** the decompensation prediction and **(b)** the LCS prediction.

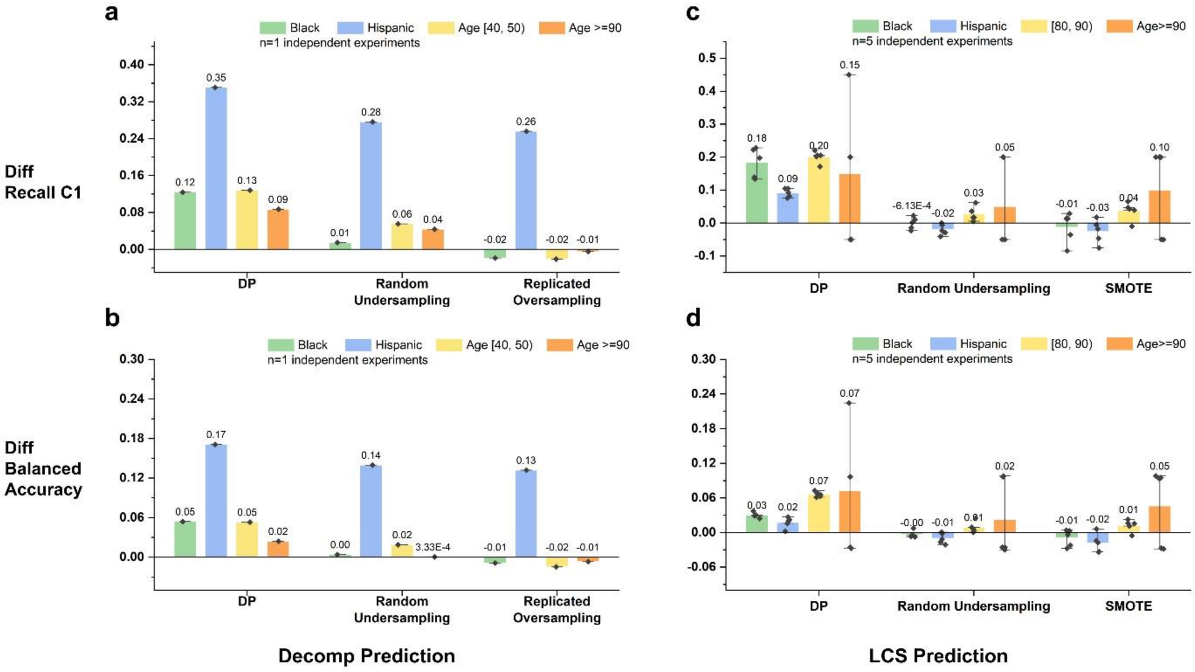

Supplementary Figure 8: DP and two representative sampling techniques (random undersampling and replicated oversampling for Decomp and random undersampling and SMOTE for LCS) performance comparison over the original model for four demographic subgroups with poor original performance. Positive values indicate performance improvement, and negative values indicate performance degradation from the original model. The error bars represent the standard error of the experiment results. **(a)** In terms of recall C1 for Decomp prediction with the MIMIC III dataset. **(b)** In terms of balanced accuracy for Decomp prediction with the MIMIC III dataset. **(c)** In terms of recall C1 for the LCS prediction with the SEER dataset. **(d)** In terms of balanced accuracy for the LCS prediction with the SEER dataset. Due to the slow decompensation computation, each decompensation prediction is executed only once.

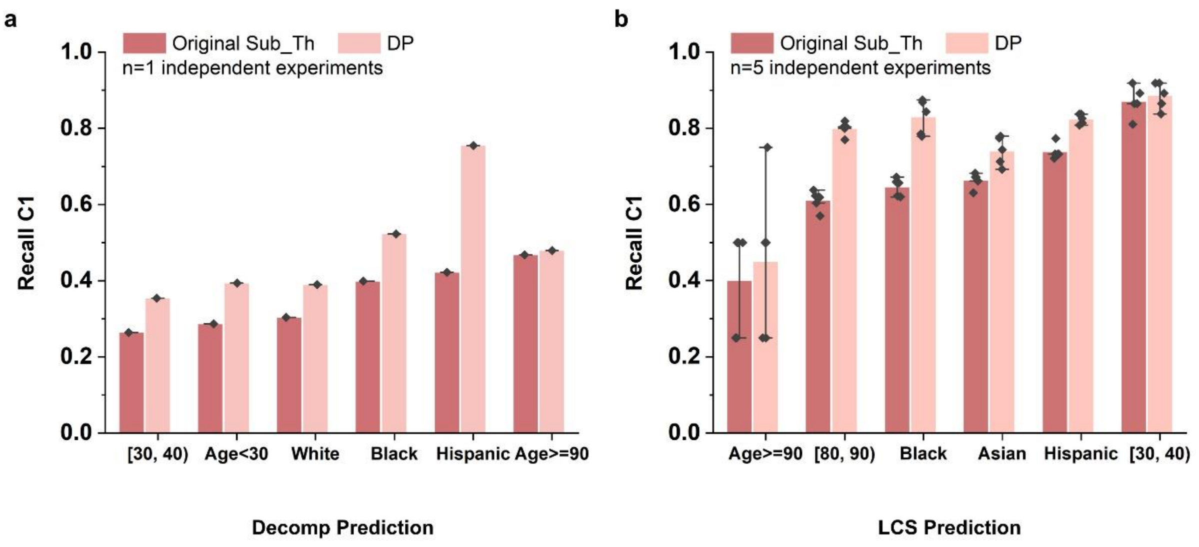

Supplementary Figure 9: Performance of DP and subgroup-threshold-based original model in terms of minority class recall for decompensation prediction and 5-year lung cancer survivability (LCS) prediction. Darker red color represents the original model performance using subgroup optimized threshold and the lighter red color represents DP performance. The error bars represent the standard error of the experiment results. Model performance comparison for **(a)** Decomp prediction task and **(b)** LCS prediction task of 6 different racial or age subgroups. For the LCS task, the standard deviation values for DP are less than 0.04, with the exception of the age 90+ group (0.187). Due to the computation complexity, we only conducted the decompensation experiments once.

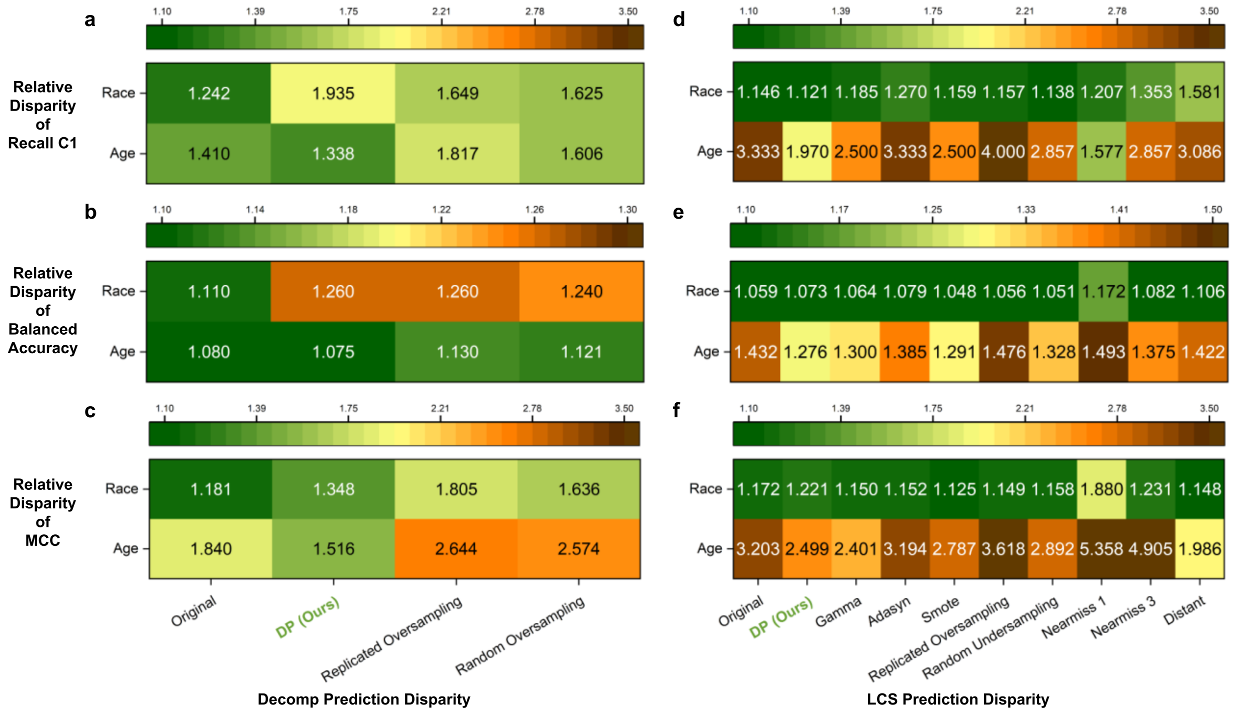

Supplementary Figure 10: Relative disparity among racial and age groups under various sampling conditions, including DP and the original machine learning model without any bias correction. The relative disparity of MIMIC III Decomp prediction in terms of **(a)** minority class recall, **(b)** balanced accuracy, and **(c)** Matthews Correlation Coefficient (MCC). The relative disparity of SEER LCS prediction in terms of **(d)** minority class recall, **(e)** balanced accuracy, and **(f)** Matthews Correlation Coefficient (MCC).

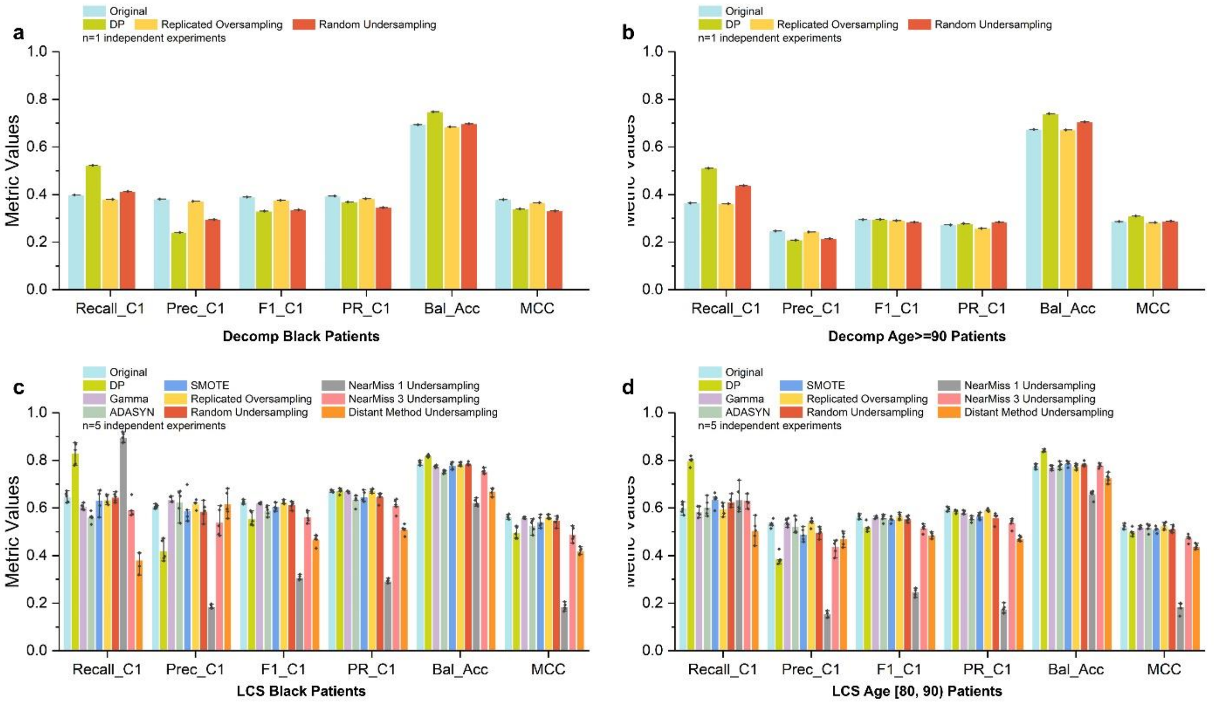

Supplementary Figure 11: Decompensation prediction and 5-year lung cancer survivability (LCS) prediction under various sampling conditions, including DP and the original machine learning model without any sampling, in terms of minority class recall, precision, F1 score, AUC-PR, balanced accuracy, and Matthews Correlation Coefficient (MCC). The error bars represent the standard error of the experiment results. Prediction results from the original model and different sampling models for **(a)** Black patients and **(b)** age>=90 patients in the Decomp prediction with the MIMIC III dataset. Prediction results from the original model and different sampling models for **(c)** Black patients and **(d)** age [80, 90) patients in the LCS prediction with the SEER dataset. Due to the slow decompensation computation, each prediction is executed only once.

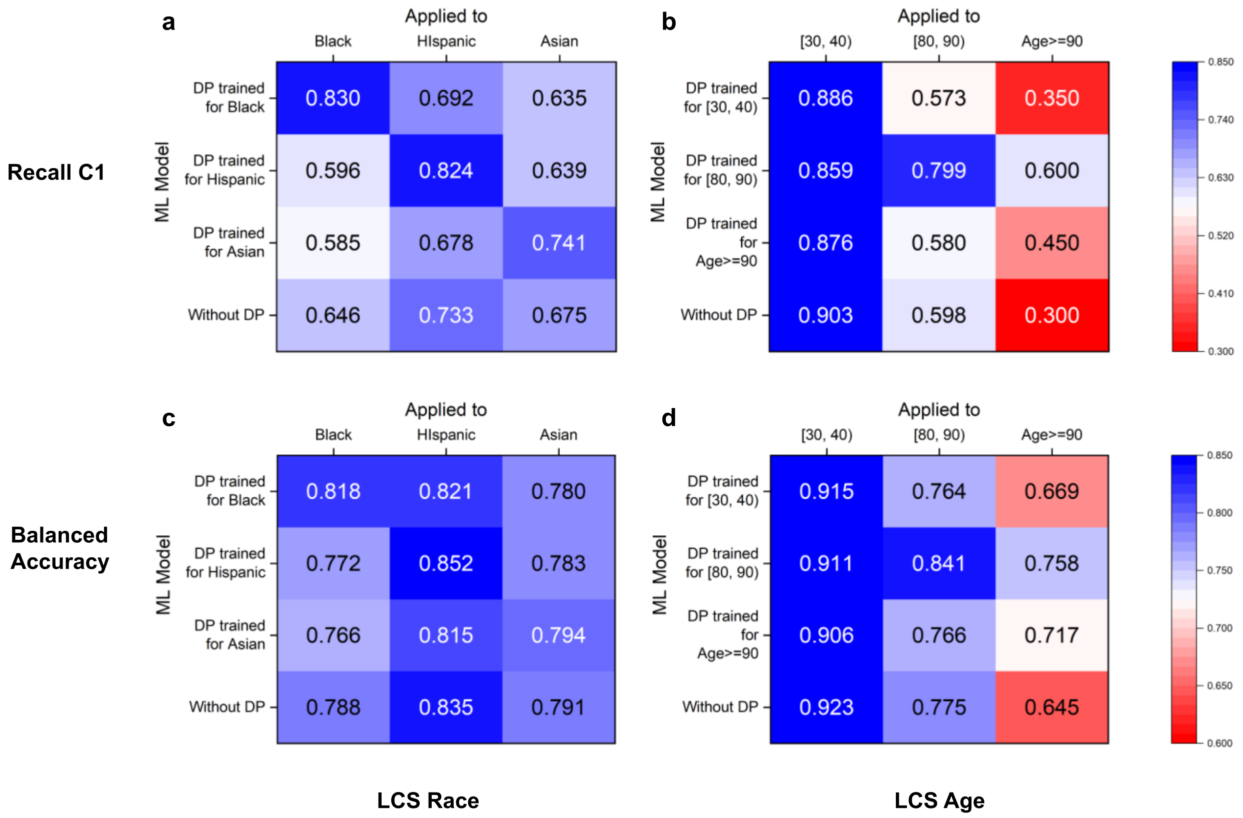

Supplementary Figure 12: DP's cross-group performance under various race and age settings for recall C1 and balanced accuracy for the LCS prediction. In subfigures, each row represents a model trained for a specific subgroup using DP. Each column represents a subgroup that a model is evaluated on. The values on the diagonal are the performance of a matching DP model, i.e., a DP model applied to the subgroup that it is designed for. The last rows show the group's performance in the original model. To prevent overfitting, our method chooses optimal thresholds based on whole group performance. DP cross-group performance for **(a)** race subgroups and **(b)** age subgroups for the LCS prediction in terms of recall C1. DP cross-group performance for **(c)** race subgroups and **(d)** age subgroups for the LCS prediction in terms of balanced accuracy.

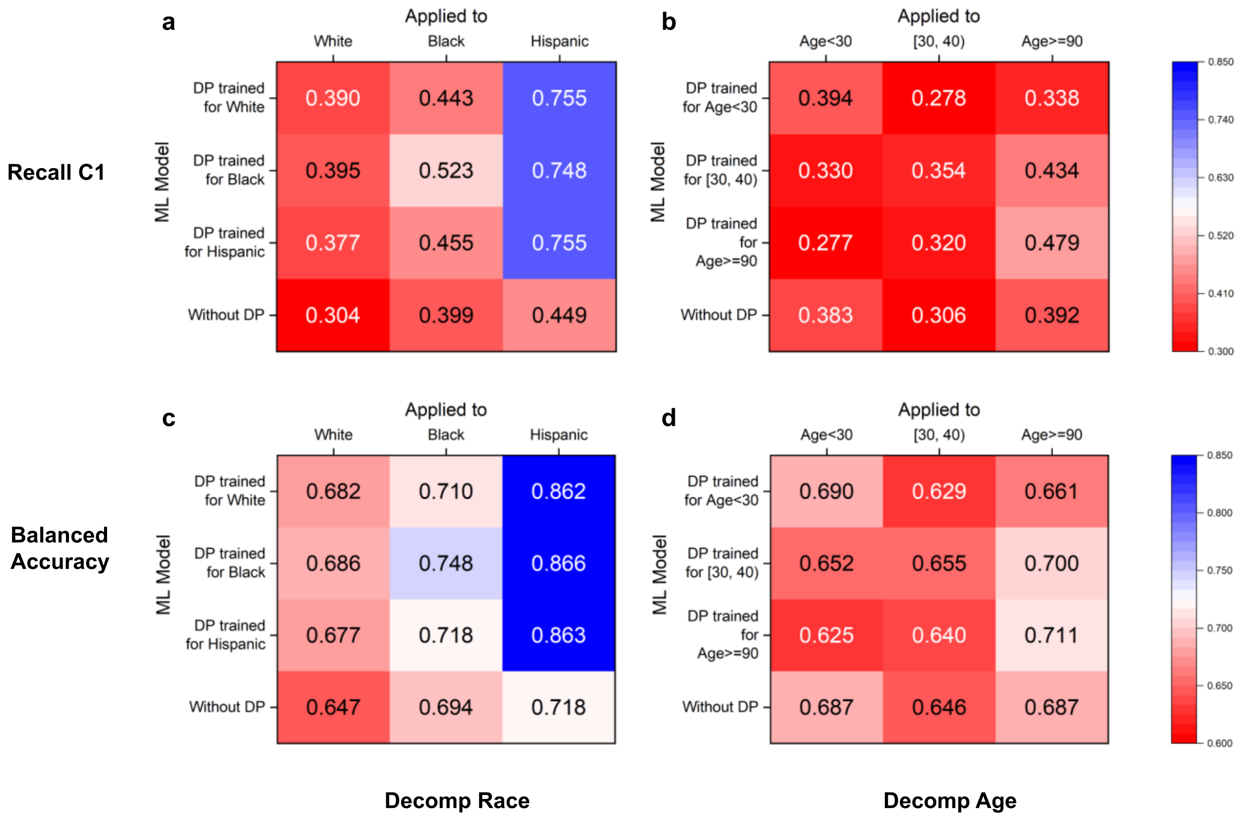

Supplementary Figure 13: DP's cross-group performance under various race and age settings for recall C1 and balanced accuracy for the decompensation prediction. In subfigures, each row corresponds to a DP model trained for a specific subgroup. Each column represents a subgroup that a model is evaluated on. The values on the diagonal are the performance of a matching DP model, i.e., a DP model applied to the subgroup that it is designed for. The last rows show the group's performance in the original model. To prevent overfitting, our method chooses optimal thresholds based on whole group performance, as opposed to the (small) minority groups in the validation sets. DP cross-group performance for **(a)** race subgroups and **(b)** age subgroups for the decompensation prediction in terms of recall C1. DP cross-group performance for **(c)** race subgroups and **(d)** age subgroups for the decompensation prediction in terms of balanced accuracy.

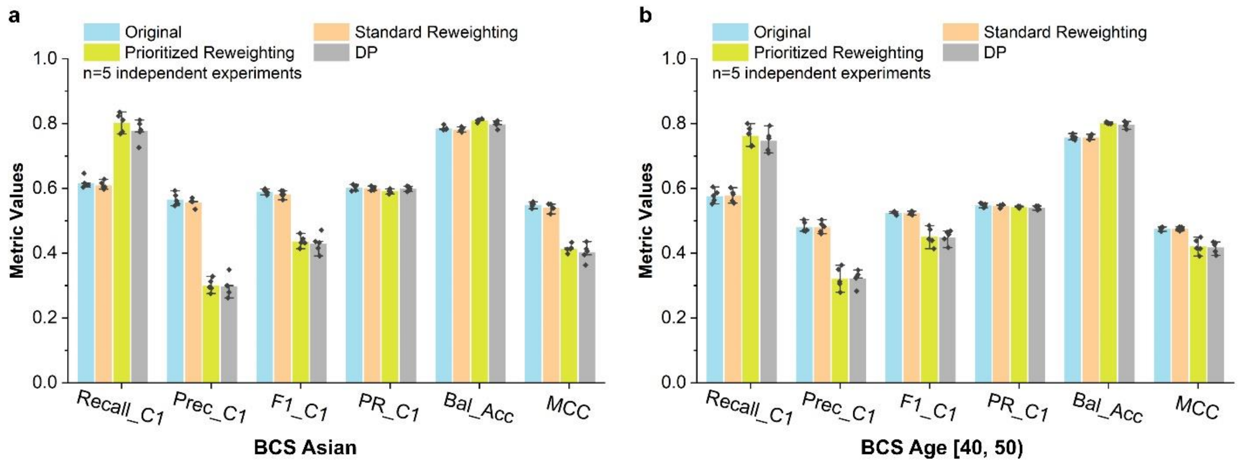

Supplementary Figure 14: Performance comparison of the original model (without bias correction), standard reweighting, prioritized reweighting, and DP for (a) BCS Asian patients and (b) BCS [40, 50) patients. The error bars represent the standard error of the experiment results. In prioritized reweighting, we dynamically increase the weight of minority class (C1) samples of selected subgroups from 1 to 20 and select the best model using the same procedure as DP.

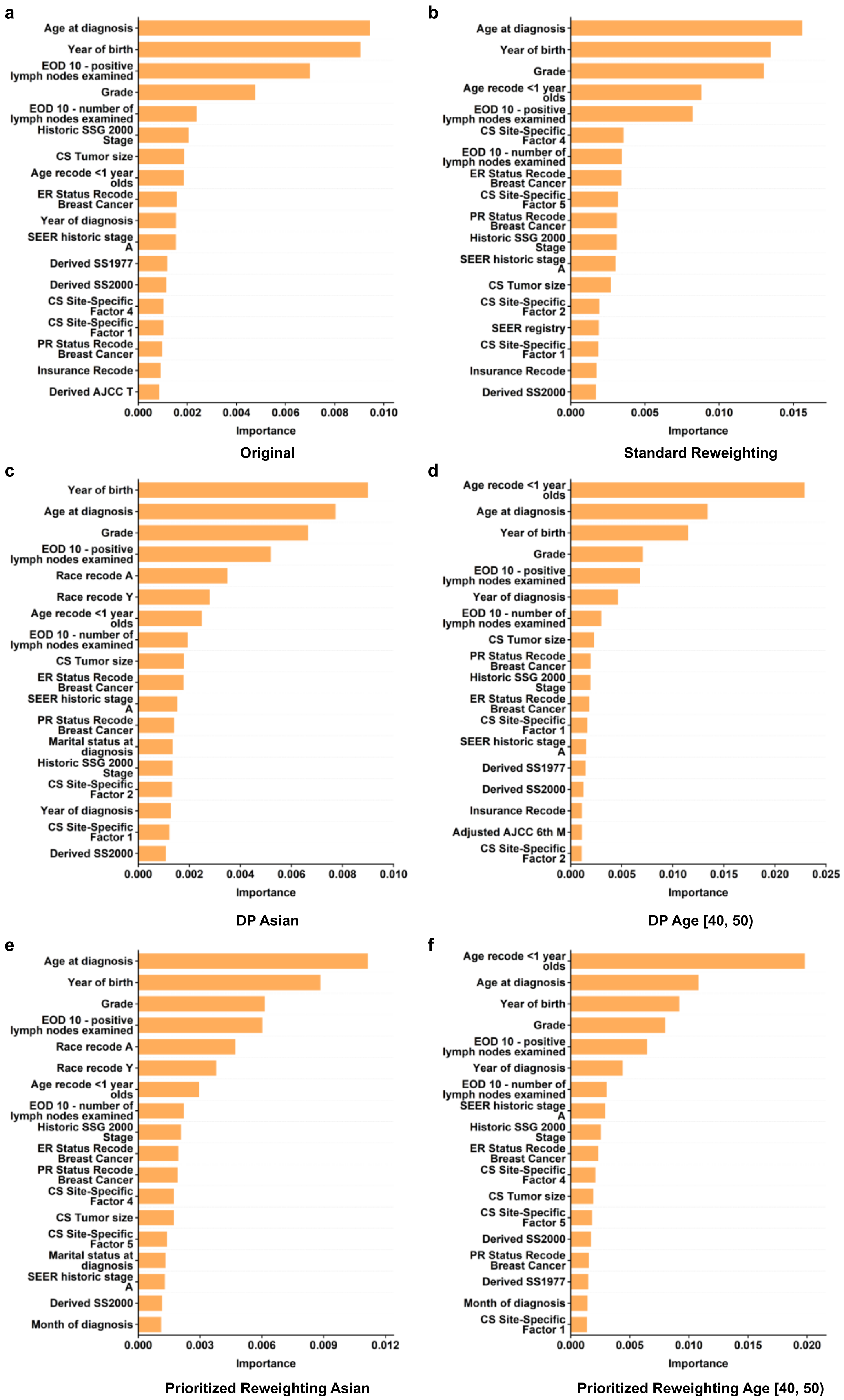
a
Age at diagnosis
Year of birth
EOD 10 - positive lymph nodes examined
Grade
EOD 10 - number of lymph nodes examined
Historic SSG 2000 Stage
CS Tumor size
Age recode <1 year olds
ER Status Recode Breast Cancer
Year of diagnosis
SEER historic stage A
Derived SS1977
Derived SS2000
CS Site-Specific Factor 4
CS Site-Specific Factor 1
PR Status Recode Breast Cancer
Insurance Recode
Derived AJCC T
0.000 0.002 0.004 0.006 0.008 0.010
Importance
Original
b
Age at diagnosis
Year of birth
Grade
Age recode <1 year olds
EOD 10 - positive lymph nodes examined
CS Site-Specific Factor 4
EOD 10 - number of lymph nodes examined
ER Status Recode Breast Cancer
CS Site-Specific Factor 5
PR Status Recode Breast Cancer
Historic SSG 2000 Stage
SEER historic stage A
CS Tumor size
CS Site-Specific Factor 2
SEER registry
CS Site-Specific Factor 1
Insurance Recode
Derived SS2000
0.000 0.005 0.010 0.015
Importance
Standard Reweighting
c
Year of birth
Age at diagnosis
Grade
EOD 10 - positive lymph nodes examined
Race recode A
Race recode Y
Age recode <1 year olds
EOD 10 - number of lymph nodes examined
CS Tumor size
ER Status Recode Breast Cancer
SEER historic stage A
PR Status Recode Breast Cancer
Marital status at diagnosis
Historic SSG 2000 Stage
CS Site-Specific Factor 2
Year of diagnosis
CS Site-Specific Factor 1
Derived SS2000
0.000 0.002 0.004 0.006 0.008 0.010
Importance
DP Asian
d
Age recode <1 year olds
Age at diagnosis
Year of birth
Grade
EOD 10 - positive lymph nodes examined
Year of diagnosis
EOD 10 - number of lymph nodes examined
CS Tumor size
PR Status Recode Breast Cancer
Historic SSG 2000 Stage
ER Status Recode Breast Cancer
CS Site-Specific Factor 1
SEER historic stage A
Derived SS1977
Derived SS2000
Insurance Recode
Adjusted AJCC 6th M
CS Site-Specific Factor 2
0.000 0.005 0.010 0.015 0.020 0.025
Importance
DP Age [40, 50)
e
Age at diagnosis
Year of birth
Grade
EOD 10 - positive lymph nodes examined
Race recode A
Race recode Y
Age recode <1 year olds
EOD 10 - number of lymph nodes examined
Historic SSG 2000 Stage
ER Status Recode Breast Cancer
PR Status Recode Breast Cancer
CS Site-Specific Factor 4
CS Tumor size
CS Site-Specific Factor 5
Marital status at diagnosis
SEER historic stage A
Derived SS2000
Month of diagnosis
0.000 0.003 0.006 0.009 0.012
Importance
Prioritized Reweighting Asian
f
Age recode <1 year olds
Age at diagnosis
Year of birth
Grade
EOD 10 - positive lymph nodes examined
Year of diagnosis
EOD 10 - number of lymph nodes examined
SEER historic stage A
Historic SSG 2000 Stage
ER Status Recode Breast Cancer
CS Site-Specific Factor 4
CS Tumor size
CS Site-Specific Factor 5
Derived SS2000
PR Status Recode Breast Cancer
Derived SS1977
Month of diagnosis
CS Site-Specific Factor 1
0.000 0.005 0.010 0.015 0.020
Importance
Prioritized Reweighting Age [40, 50)

Supplementary Figure 15: SHAP-avg feature importance of different BCS experiments. Original stands for the original machine learning model without any bias correction. DP stands for our Double Prioritized sampling method. Standard reweighting and prioritized reweighting are described in the Methods Section. In SHAP-avg, the SHAP importance of columns representing the same variable is averaged. The AJCC (American Joint Committee on Cancer) staging system is a system used to describe most types of cancer. SSG stands for the summary stage. ICD describes primary tumor site/type. PR and ER status represent a combination of a tumor marker and a site factor. Detailed variable and recode definitions can be found on the SEER website (https://seer.cancer.gov/data-software/documentation/seerstat/nov2016/). Feature importance for BCS prediction in (a) original model, (b) standard reweighting model, (c) DP model for Asian patients, (d) DP model for age [40, 50) patients, (e) prioritized reweighting model for Asian patients, and (f) prioritized reweighting model for age [40, 50) patients.

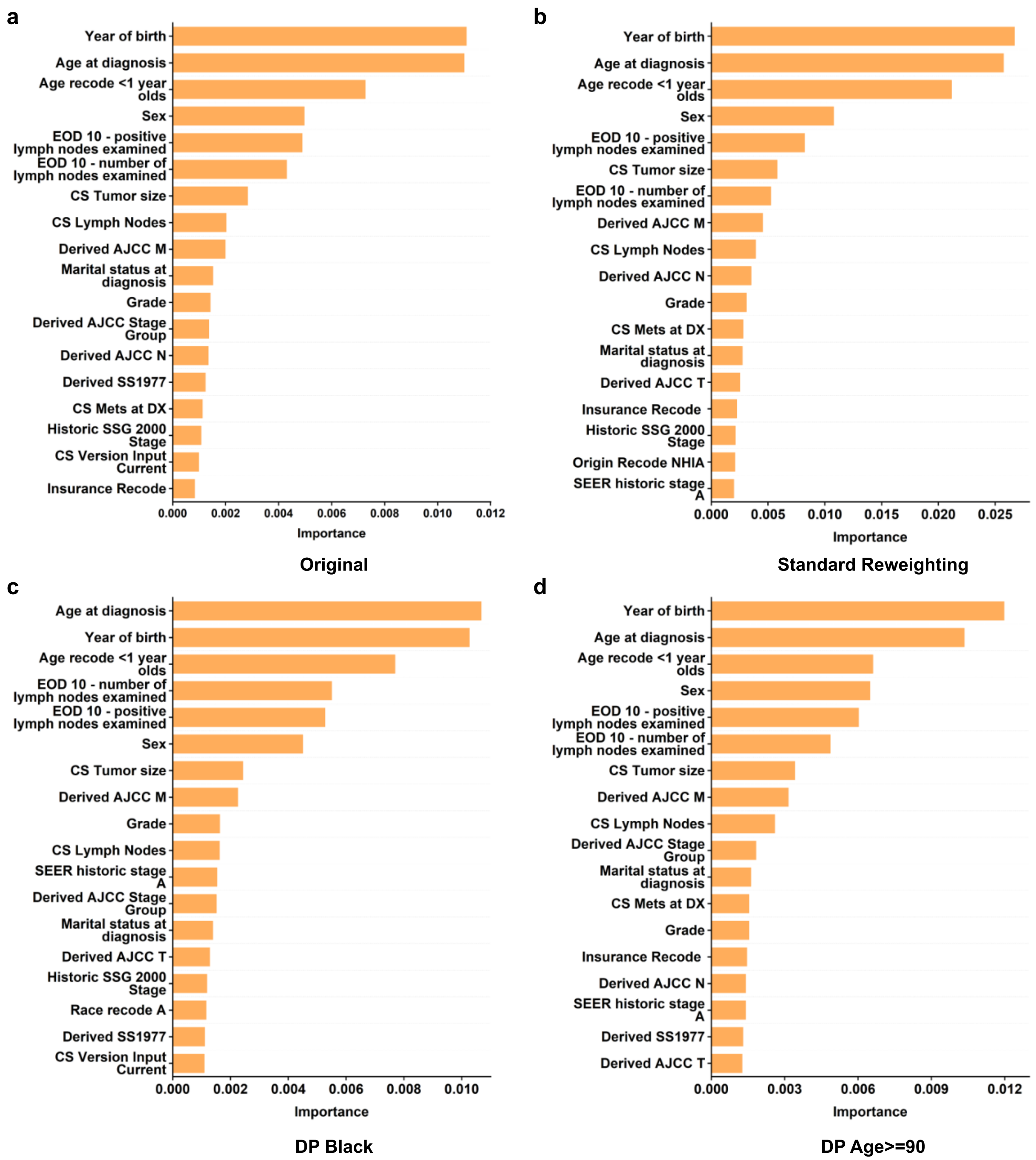

Supplementary Figure 16: SHAP-avg feature importance of different LCS experiments. Original represents the original machine learning without any bias correction. DP stands for our Double Prioritized sampling method. Standard reweighting is described in the Methods section. In SHAP-avg, the importance of columns representing the same variable is averaged. The AJCC (American Joint Committee on Cancer) staging system is a system used to describe most types of cancer. SSG stands for the summary stage. ICD describes primary tumor site/type. CS Mets at DX provides information on distant metastasis, describing the extent of the disease. Detailed variable and recode definitions can be found on the SEER website (https://seer.cancer.gov/data-software/documentation/seerstat/nov2016/). Feature importance for LCS prediction in **(a)** original model, **(b)** standard reweighting model, **(c)** DP model for Black patients, and **(d)** DP model for age>=90 patients.

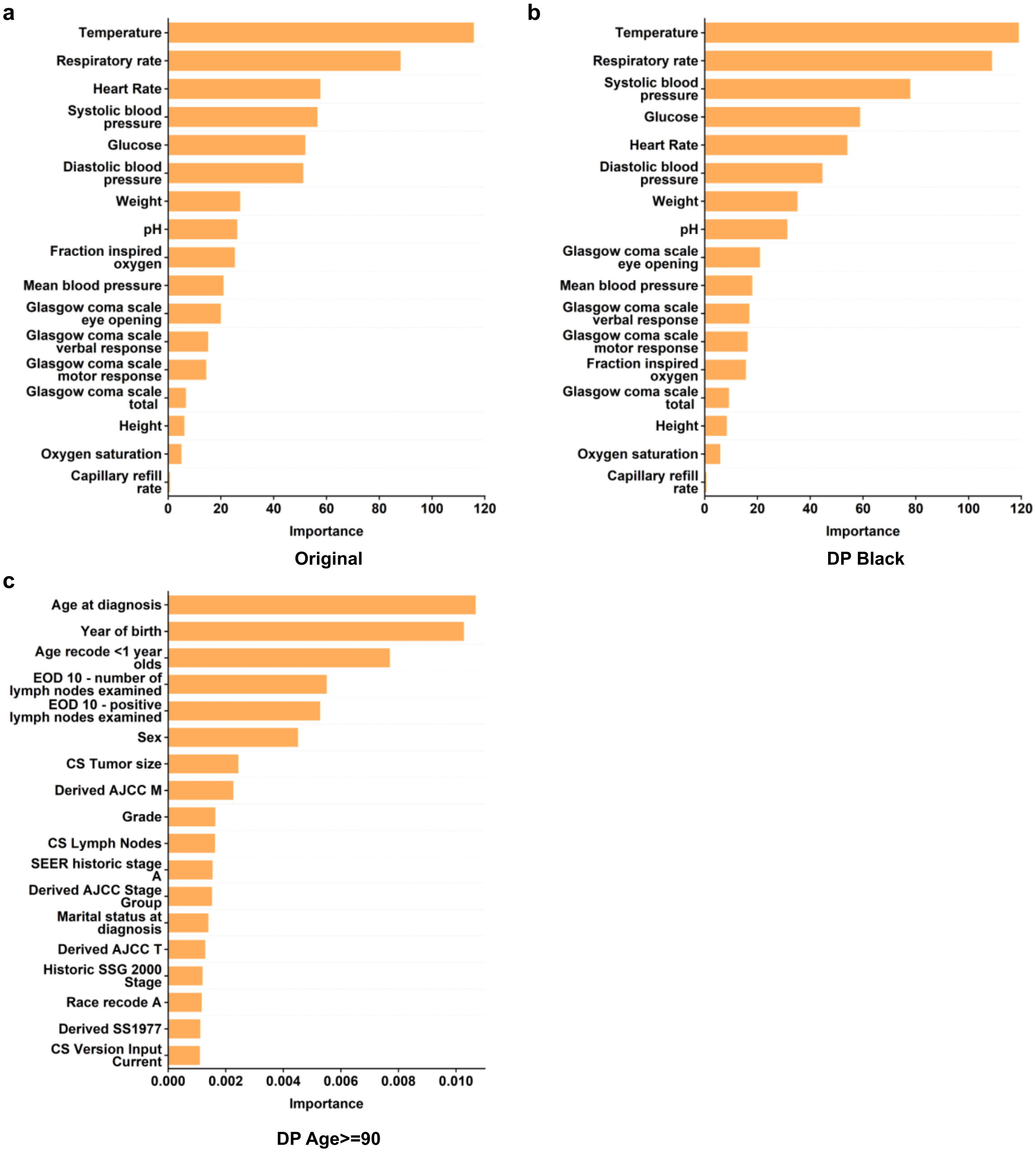

Supplementary Figure 17: SHAP-avg feature importance of different IHM experiments. Original stands for the original machine learning model without any bias correction. DP stands for our Double Prioritized sampling method. In SHAP-avg, the importance of columns representing the same variable is averaged. Feature importance for IHM prediction in **(a)** original model, **(b)** DP model for Black patients, and **(c)** DP model for age>=90 patients.

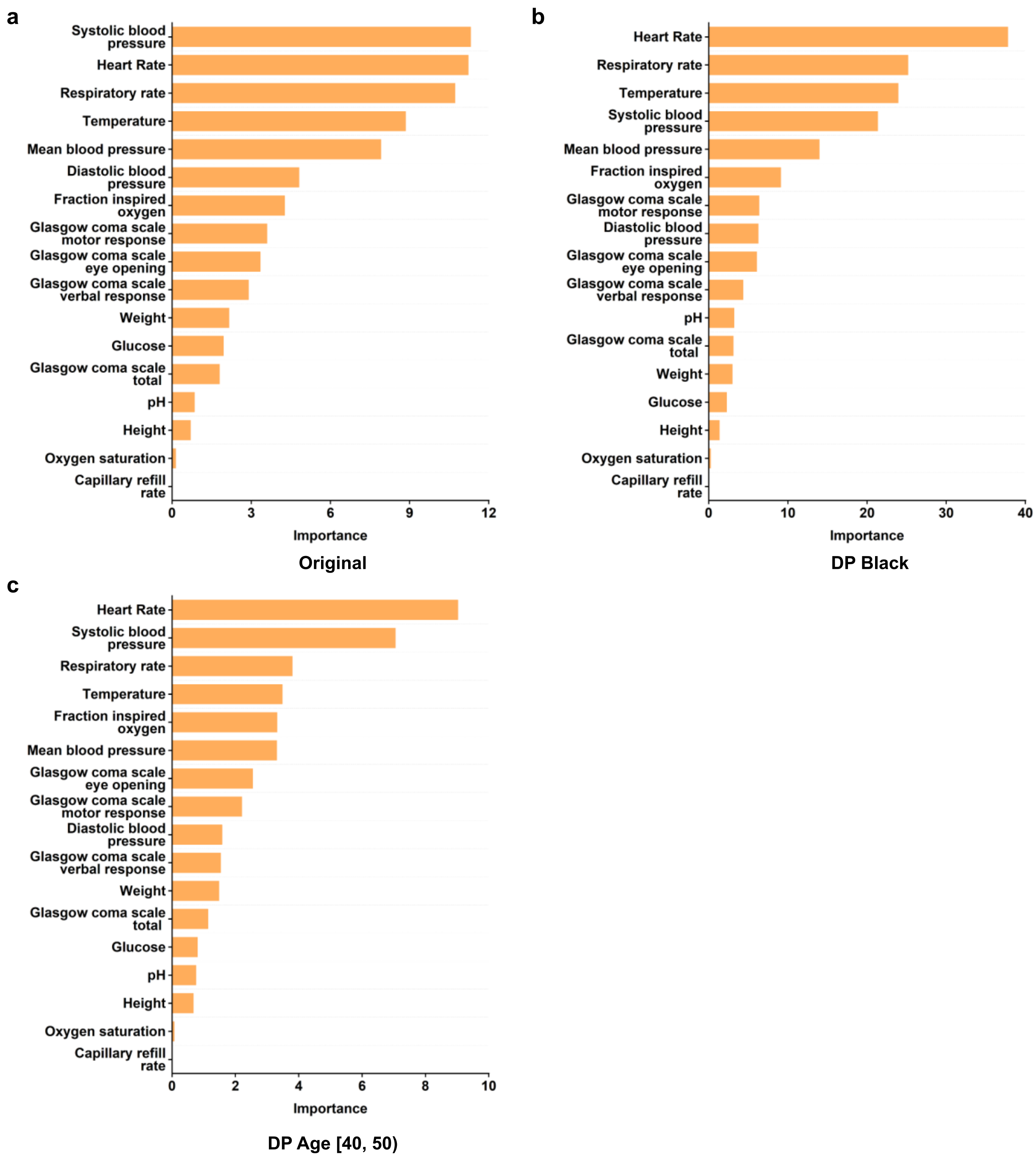

Supplementary Figure 18: SHAP-avg feature importance of different decompensation experiments. Original stands for the original machine learning model without any bias correction. DP stands for our Double Prioritized sampling method. In SHAP-avg, the importance of columns representing the same variable is averaged. Feature importance for the decompensation prediction in **(a)** original model, **(b)** DP model for Black patients, and **(c)** DP model for age [40, 50) patients.

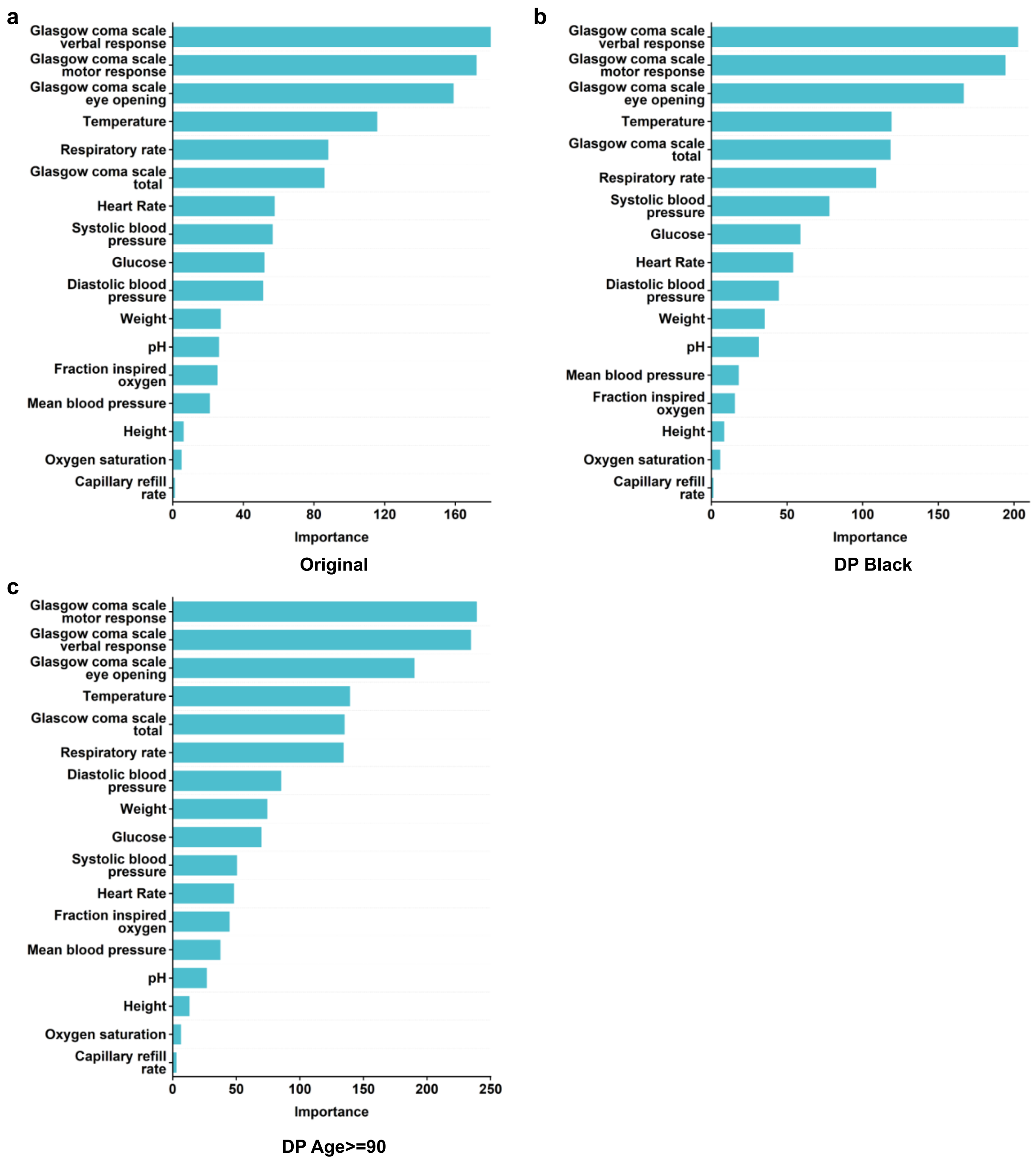

Supplementary Figure 19: SHAP-sum feature importance of different IHM experiments. Original stands for the original machine learning model without any bias correction. DP stands for our Double Prioritized sampling method. In SHAP-sum, the importance of columns representing the same variable is summed up. Feature importance for the IHM prediction in **(a)** original model, **(b)** DP model for Black patients, and **(c)** DP model for age>=90 patients.

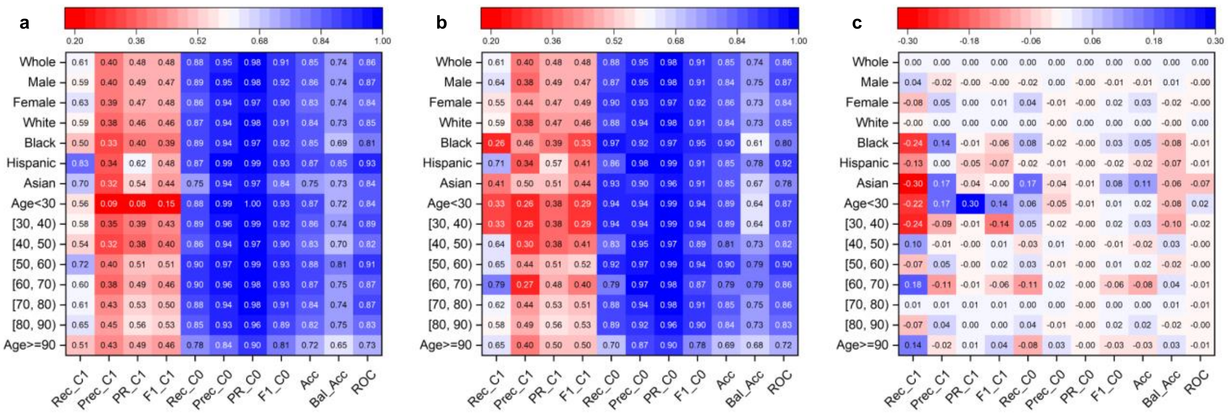

Supplementary Figure 20: In-hospital mortality prediction performance of the original model with (a) whole group calibration, (b) subgroup calibration, and (c) difference in the performance between whole group and subgroup calibration. A positive value means subgroup calibration improves the performance. Rec_C1, Prec_C1, PR_C1, F1_C1, Rec_C0, Prec_C0, PR_C0, F1_C0, Acc, Bal_Acc, ROC stand for Recall Class 1, Precision Class 1, Area Under the Precision-Recall Curve Class 1, F1 score Class 1, Recall Class 0, Precision Class 0, Area Under the Precision-Recall Curve Class 0, F1 score Class 0, Accuracy, Balanced Accuracy, Area under the ROC Curve, respectively.

Supplementary Table 1: Learning parameters for four prediction models. BCS stands for breast cancer survivability. IHM stands for in-hospital mortality. LCS stands for lung cancer survivability. Decomp stands for decompensation. ANN stands for the artificial neural network.

Learning Parameter	BCS Prediction	IHM Prediction	LCS Prediction	Decomp Prediction
Hidden layers	(20, 20)	(16, 16)	(20, 20)	(128)
ANN	MLP	LSTM	MLP	LSTM
Learning Rate	0.001	0.001	0.001	0.001
Optimizer	adam	adam	adam	adam
Dropout	0.1	0.3	0.1	0.0

For the IHM prediction task with MIMIC III datasets, training involves 100 epochs or stops early based on validation performance. For DP, we run for 50 epochs up to 20 additional units. For the Decomp prediction task with MIMIC III datasets, training involves 50 epochs or stops early based on validation performance. For DP experiments, we run for 10 epochs up to 20 additional units. The SEER cancer dataset is smaller, thus for the cancer prediction tasks, we run 25 epochs for all experiments. Each epoch produces a machine learning model; to choose the final model, we first identify the top three models based on balanced accuracy and then select the one with the highest precision-recall curve value of the minority class (denoted as PR_C1). For the SEER dataset, 80% is used for training, 10% for validation, and 10% for testing. For MIMIC III, the percentages are 70% for training, 15% for validation, and 15% for testing.

Supplementary Table 2: Performance comparison of standard reweighting with the original model and DP. Performance of the original model, applying DP, and applying standard reweighting for the BCS prediction and LCS prediction. For BCS, the minority class (C1) has a weight of 3.94 and the majority class (C0) has a weight of 0.57. For LCS, the minority class (C1) has a weight of 3.12 and the majority class (C0) has a weight of 0.60. Orig refers to the original model. SR stands for standard reweighting.

	**Recall C1**			**F1 C1**			**Balanced Accuracy**		
	Orig	DP	SR	Orig	DP	SR	Orig	DP	SR
**BCS Asian**	0.617	0.778	0.610	0.590	0.429	0.582	0.785	0.798	0.781
**BCS Age [40, 50)**	0.577	0.747	0.577	0.524	0.450	0.524	0.758	0.797	0.758
**LCS Black**	0.646	0.830	0.634	0.625	0.555	0.626	0.788	0.818	0.787
**LCS Age>=90**	0.300	0.450	0.300	0.269	0.327	0.258	0.645	0.717	0.644

Supplementary Table 3: Summary of cross-race and cross-age-group results in the IHM, BCS, LCS, and Decomp tasks. A key case refers to that the matching DP models (i.e., sample enrichment matches the test group's demographics) achieve the highest recall C1 performance.

**Task**	**No. of Key Cases**	**Race (No.)**	**Age Group (No.)**	**Figure Number**
**IHM**	3 (out of 6)	Black (1)	<30, 90+ (2)	Supp. Fig. 4
**BCS**	5 (out of 6)	Black, Hispanic, Asian (3)	<30, [30, 40) (2)	Fig. 8
**LCS**	4 (out of 6)	Black, Hispanic, Asian (3)	[80, 90) (1)	Supp. Fig. 12
**Decomp**	4 (out of 6)	Black (1)	<30, [30, 40), 90+ (3)	Supp. Fig. 13
**Total**	**16 (Out of 24)**	**8**	**8**	**--**

Supplementary Table 4: Performance of MLP models using different structures. The performance of MLP models on the BCS and LCS tasks. We evaluate 3 different numbers of layers, 3 different numbers of neurons per layer, and 3 different dropout rates, generating 27 models in total for each task. The results are comparable among the models. The table shows the subgroup performance of the default model (2 layers with 20 neurons, 0.1 dropout rate) compared with two other models (5 layers with 30 neurons, 0.2 dropout rate and 10 layers with 50 neurons, 0.3 dropout rate).

	**Recall C1**			**F1 C1**			**Balanced Accuracy**		
	**2-20-0.1 (default)**	**5-30-0.2**	**10-50-0.3**	**2-20-0.1 (default)**	**5-30-0.2**	**10-50-0.3**	**2-20-0.1 (default)**	**5-30-0.2**	**10-50-0.3**
**BCS Asian**	0.617	0.627	0.643	0.590	0.584	0.591	0.785	0.788	0.795
**BCS Age [40, 50)**	0.577	0.571	0.607	0.524	0.518	0.514	0.758	0.755	0.767
**LCS Black**	0.646	0.644	0.653	0.625	0.622	0.631	0.788	0.787	0.792
**LCS Age>=90**	0.300	0.250	0.300	0.269	0.242	0.310	0.645	0.620	0.646

Supplementary Table 5: Relative disparity of MLP models using different structures. The relative disparity among subgroups for the BCS and LCS tasks are shown, including the disparity of the default model (2 layers with 20 neurons, 0.1 dropout rate) compared with two other models (5 layers with 30 neurons, 0.2 dropout rate and 10 layers with 50 neurons, 0.3 dropout rate).

	**Recall C1**			**Balanced Accuracy**		
	**2-20-0.1 (default)**	**5-30-0.2**	**10-50-0.3**	**2-20-0.1 (default)**	**5-30-0.2**	**10-50-0.3**
**BCS Race**	1.205	1.237	1.237	1.044	1.050	1.047
**BCS Age**	1.580	1.574	1.488	1.139	1.129	1.118
**LCS Race**	1.146	1.138	1.127	1.059	1.056	1.052
**LCS Age**	3.333	4.000	3.333	1.432	1.485	1.429
