# Supplementary material for: Subpopulation-specific machine learning prognosis for underrepresented patients with double prioritized bias correction
Source: Commun Med (Lond). 2022 Sep 1;2:111. doi: 10.1038/s43856-022-00165-w (PMC9436942; doi:10.1038/s43856-022-00165-w)
Supplement: Supplementary file 3 — Description of Additional Supplementary Files [file 43856_2022_165_MOESM3_ESM.pdf]

Description of Additional Supplementary Files

File Name: Supplementary Data 1

Description: All source data for main figures
