# Supplementary material for: Subpopulation-specific machine learning prognosis for underrepresented patients with double prioritized bias correction
Source: Commun Med (Lond). 2022 Sep 1;2:111. doi: 10.1038/s43856-022-00165-w (PMC9436942; doi:10.1038/s43856-022-00165-w)
Supplement: Supplementary file 4 — Reporting Summary [file 43856_2022_165_MOESM4_ESM.pdf]

nature research

Corresponding author(s): Danfeng (Daphne) Yao

Last updated by author(s): Jun 10, 2022

Reporting Summary

Nature Research wishes to improve the reproducibility of the work that we publish. This form provides structure for consistency and transparency in reporting. For further information on Nature Research policies, see our Editorial Policies and the Editorial Policy Checklist.

Statistics

For all statistical analyses, confirm that the following items are present in the figure legend, table legend, main text, or Methods section.

n/a	Confirmed	
☐	☒	The exact sample size (*n*) for each experimental group/condition, given as a discrete number and unit of measurement
☐	☒	A statement on whether measurements were taken from distinct samples or whether the same sample was measured repeatedly
☒	☐	The statistical test(s) used AND whether they are one- or two-sided<br>*Only common tests should be described solely by name; describe more complex techniques in the Methods section.*
☐	☒	A description of all covariates tested
☒	☐	A description of any assumptions or corrections, such as tests of normality and adjustment for multiple comparisons
☐	☒	A full description of the statistical parameters including central tendency (e.g. means) or other basic estimates (e.g. regression coefficient) AND variation (e.g. standard deviation) or associated estimates of uncertainty (e.g. confidence intervals)
☒	☐	For null hypothesis testing, the test statistic (e.g. *F*, *t*, *r*) with confidence intervals, effect sizes, degrees of freedom and *P* value noted<br>*Give P values as exact values whenever suitable.*
☒	☐	For Bayesian analysis, information on the choice of priors and Markov chain Monte Carlo settings
☐	☒	For hierarchical and complex designs, identification of the appropriate level for tests and full reporting of outcomes
☒	☐	Estimates of effect sizes (e.g. Cohen's *d*, Pearson's *r*), indicating how they were calculated

Our web collection on statistics for biologists contains articles on many of the points above.

Software and code

Policy information about availability of computer code

Data collection	No software was used
Data analysis	Open source programming language Python (version 3.8) is used for data analysis

For manuscripts utilizing custom algorithms or software that are central to the research but not yet described in published literature, software must be made available to editors and reviewers. We strongly encourage code deposition in a community repository (e.g. GitHub). See the Nature Research guidelines for submitting code & software for further information.

Data

Policy information about availability of data

All manuscripts must include a data availability statement. This statement should provide the following information, where applicable:

- Accession codes, unique identifiers, or web links for publicly available datasets
- A list of figures that have associated raw data
- A description of any restrictions on data availability

The MIMIC III and SEER data used in this study are not publicly downloadable but can be easily requested and obtained from their original sites. Parties interested in data access should visit the MIMIC III website (https://mimic.physionet.org/gettingstarted/access/) and the SEER website (https://seer.cancer.gov/data/access.html) to submit access requests. Source data for the figures are available as Supplementary Data 1.

Field-specific reporting

Please select the one below that is the best fit for your research. If you are not sure, read the appropriate sections before making your selection.

☒ Life sciences ☐ Behavioural & social sciences ☐ Ecological, evolutionary & environmental sciences

For a reference copy of the document with all sections, see nature.com/documents/nr-reporting-summary-flat.pdf

Life sciences study design

All studies must disclose on these points even when the disclosure is negative.

Sample size	No sample size calculation was performed. We used samples from openly available MIMIC III and SEER clinical databases.
Data exclusions	The exclusion criteria were pre-established. For MIMIC III, patients data were excluded if the patient is less than 18 years old, if the patient transfers from ICU, etc. (Harutyunyan 2019). For SEER, patients data were excluded if ICD-O-3 is not breast/lung cancer, not diagnosed from 2004 to 2009, etc (Hegselmann 2018).
Replication	We provide the website link of available MIMIC III and SEER database, GitHub link for prediction and analysis code.
Randomization	This is not relevant to our study. We directly use patient's information from available renowned clinical datasets.
Blinding	This is not relevant to our study. We directly use patient's information from available renowned clinical datasets.

Reporting for specific materials, systems and methods

We require information from authors about some types of materials, experimental systems and methods used in many studies. Here, indicate whether each material, system or method listed is relevant to your study. If you are not sure if a list item applies to your research, read the appropriate section before selecting a response.

Materials & experimental systems

n/a	Involved in the study	
☒	☐	Antibodies
☒	☐	Eukaryotic cell lines
☒	☐	Palaeontology and archaeology
☒	☐	Animals and other organisms
☒	☐	Human research participants
☒	☐	Clinical data
☒	☐	Dual use research of concern

Methods

n/a	Involved in the study	
☒	☐	ChIP-seq
☒	☐	Flow cytometry
☒	☐	MRI-based neuroimaging
